# Deep brain stimulation of the ventral striatum increases BDNF in the fear extinction circuit

**DOI:** 10.3389/fnbeh.2013.00102

**Published:** 2013-08-08

**Authors:** Fabricio H. Do-Monte, Jose Rodriguez-Romaguera, Luis E. Rosas-Vidal, Gregory J. Quirk

**Affiliations:** Departments of Psychiatry and Anatomy and Neurobiology, University of Puerto Rico School of MedicineSan Juan, Puerto Rico

**Keywords:** anxiety disorders, obsessive compulsive disorder, prefrontal cortex, amygdala, Fos, high-frequency stimulation, fear expression

## Abstract

Deep brain stimulation (DBS) of the ventral capsule/ventral striatum (VC/VS) reduces the symptoms of treatment-resistant obsessive compulsive disorder (OCD), and improves response to extinction-based therapies. We recently reported that DBS-like stimulation of a rat homologue of VC/VS, the dorsal-VS, reduced conditioned fear and enhanced extinction memory (Rodriguez-Romaguera et al., 2012). In contrast, DBS of the ventral-VS had the opposite effects. To examine possible mechanisms of these effects, we assessed the effects of VS DBS on the expression of the neural activity marker Fos and brain-derived neurotrophic factor (BDNF), a key mediator of extinction plasticity in prefrontal-amygdala circuits. Consistent with decreased fear expression, DBS of dorsal-VS increased Fos expression in prelimbic and infralimbic prefrontal cortices and in the lateral division of the central nucleus of amygdala, an area that inhibits amygdala output. Consistent with improved extinction memory, we found that DBS of dorsal-VS, but not ventral-VS, increased neuronal BDNF expression in prelimbic and infralimbic prefrontal cortices. These rodent findings are consistent with the idea that clinical DBS of VC/VS may augment fear extinction through an increase in BDNF expression.

## Introduction

Deep brain stimulation (DBS) of the ventral capsule/ventral striatum (VC/VS) reduces the symptoms of refractory obsessive compulsive disorder OCD, (Denys et al., 2010; Greenberg et al., 2010), but little is known about the mechanisms. Many OCD compulsions consist of avoidance of stimuli interpreted as threatening (Pietrefesa and Coles, 2009). Avoidance behaviors persist in the absence of danger, suggesting a deficit in extinction of fear (Rasmussen and Eisen, 1992; Milad et al., 2013). We recently observed in rats that DBS-like high frequency stimulation of the VS, a rodent homologue of the VC/VS, either enhanced or weakened extinction of fear, depending on the specific site within the VS (Rodriguez-Romaguera et al., 2012). DBS of the dorsal portion of the VS (dorsal-VS) reduced fear expression and enhanced extinction memory, whereas DBS of the ventral portion of the VS (ventral-VS) impaired extinction. The opposite effects of DBS at these VS sites offer a unique opportunity to understand the mechanisms of DBS in extinction. For example, DBS of dorsal-VS, but not ventral-VS, increased expression of the plasticity marker pERK in prefrontal and amygdala regions associated with extinction (Rodriguez-Romaguera et al., 2012).

While induction of plasticity by DBS is consistent with enhancement of extinction memory, it tells us little about the mechanisms involved. It is well established that BDNF is a key mediator of synaptic plasticity in fear circuits (see Monfils et al., 2007; Andero and Ressler, 2012 for reviews). BDNF in the basolateral amygdala (BLA) and the infralimbic (IL) prefrontal cortex have been associated with extinction learning (Chhatwal et al., 2006; Bredy et al., 2007; Peters et al., 2010; Soliman et al., 2010). Thus, BDNF could play a role in extinction-modulation by DBS. Furthermore, OCD is associated with reduced BNDF function (Maina et al., 2010; Fontenelle et al., 2012), and patients expressing a BDNF genetic polymorphism show poor response to extinction-based therapies (Fullana et al., 2012).

We therefore used an immunocytochemical approach to compare the effects of DBS of dorsal-VS vs. ventral-VS on the expression of BDNF in the medial prefrontal cortex (mPFC) and amygdala. In addition to neurons, BDNF is expressed in microglia, astrocytes, and endothelial cells (Rudge et al., 1992; Bejot et al., 2011). Therefore, to assess neuronal BDNF, we co-labeled BDNF antibodies with the neuronal marker NeuN. Furthermore, we measured the expression of Fos protein in the mPFC and amygdala as a marker of recent neuronal activity (Morgan et al., 1987; Dragunow and Faull, 1989).

## Materials and methods

### Subjects

Male Sprague-Dawley rats (*n* = 37, Harlan Laboratories) weighing ~320 g and 12–16 weeks old were used. Animals were housed individually in transparent polyethylene cages with standard environmental conditions (73–75°F and a 12 hrs light/dark cycle, light on at 7:00 A.M.) and free access to food and water. All procedures were approved by the Institutional Care and Use Committee from University of Puerto Rico School of Medicine, in compliance with the National Institutes of Health.

### Surgery

Rats were anesthetized with isofluorane inhalant gas (5%) in an induction chamber and positioned in a stereotaxic frame. Isofluorane (2–3%) was delivered through a facemask used for anesthesia maintenance throughout the surgery. Animals were stereotaxically implanted with concentric bipolar stimulating electrodes (NEX-100; Rhodes Medical Instruments) as previously described (Rodriguez-Romaguera et al., 2012). Electrodes were aimed at the dorsal portion of the ventral striatum (−6.5 mm dorsoventral from the skull surface, ±2.0 mm mediolateral from midline, and +1.2 mm anteroposterior from bregma) or at the ventral portion of the ventral striatum (−8.0 mm dorsoventral, ±2.0 mm mediolateral, and ±1.2 mm anteroposterior) (Paxinos and Watson, 1997). After surgery, rats were allowed to recover for one week before experiments initiated.

### Deep brain stimulation

Rats were initially connected to the stimulation cable in their home cage and habituated for 3 hrs on 2 consecutive days. On the following day, rats were randomly divided to receive bilateral monophasic DBS (100 µA, 0.1 ms pulse duration, 130 Hz, bipolar) continuously during 3 hrs (DBS group) or no stimulation (Sham control group). These parameters of stimulation were the same as those used to facilitate extinction in our previous study (Rodriguez-Romaguera et al., 2012). A stimulator (S88X, Grass Instruments, USA) connected to a constant-current unit (SIC-C Isolation Unit, Grass Instruments, USA) was used.

### Immunocytochemistry

Rats were deeply anesthetized with sodium pentobarbital (450 mg/Kg i.p.) immediately after receiving 3 hrs of DBS or sham stimulation in their home cages. They were perfused transcardially with 100 ml of 0.9% saline followed by 500 ml of 4% paraformaldehyde in 0.1 M phosphate buffer at pH 7.4. Brains were removed from the skull and fixed overnight in the same fixative solution. The next day, brains were transferred to a solution of 30% sucrose in 0.1 M phosphate buffer at 4°C during 48 hrs for cryoprotection. The brains were frozen and series of coronal sections (40 µm) were cut on a cryostat (CM 1850; Leica) and collected at different levels of mPFC and amygdala. Sections at the level of VS were also collected, mounted in coated-gelatin slides, stained for Nissl bodies, cover-slipped and used to determine electrode placement. Immunohistochemistry for VS sections was not assessed because previous studies have shown that DBS of VS does not induce local changes (McCracken and Grace, 2009; van Dijk et al., 2011).

For Fos immunocytochemistry experiments, alternate sections were initially blocked in a solution of 2% normal goat serum (NGS, Vector Laboratories^®^, USA) plus 0.3% triton (Triton X-100, Sigma-Aldrich^®^, USA) in 0.12 M potassium buffer saline for 1 hr, as previously described in our lab (Padilla-Coreano et al., 2012). The sections were then incubated overnight at room temperature with anti-Fos serum raised in rabbit (Ab-5, Oncogene Science^®^, USA) at a dilution of 1:20,000. The primary antiserum was localized using a variation of the avidinbiotin complex system. Sections were then incubated for 2 hrs at room temperature in a solution of biotinylated goat anti-rabbit IgG (Vector Laboratories^®^) and placed in a mixed avidin biotin horseradish peroxidase complex solution (ABC Elite Kit, Vector Laboratories^®^) for 90 min. Black immunoreactive nuclei labeled for Fos were visualized after 10 min of exposure to a chromogen solution containing 0.02% 3,3’ diaminobenzidine tetrahydrochloride with 0.3% nickel ammonium sulphate (DAB-Ni) in 0.05 M Tris buffer, pH 7.6, followed by a 10 min incubation period in a chromogen solution with glucose oxidase (10%) and D-Glucose (10%). The reaction was stopped using potassium phosphate buffered saline (PBS) (pH 7.4). Sections were mounted in coated-gelatin slides, dehydrated and cover slipped. Counter sections were collected, stained for Nissl bodies, cover slipped and used to determine the anatomical boundaries of each structure analyzed.

For BDNF immunocytochemistry, alternate sections were initially blocked in a solution of 2% normal goat serum (NGS, Vector Laboratories^®^) plus 0.3% triton (Triton X-100, Sigma-Aldrich^®^) in 0.12 M potassium buffer saline for 1 hr, as previously described (Ou et al., 2010). Sections were then incubated overnight at room temperature with sheep anti-BDNF antibody (1:200, Millipore^®^, USA) plus anti-NeuN (1:200, conjugated with rabbit polyclonal Alexa Fluor 488, Millipore^®^). The next day, slices were incubated with anti-sheep fluorescent secondary antibody (1:200, Alexa Fluor 594, Invitrogen^®^) for 2 hrs, mounted in coated-gelatin slides, dehydrated and then cover-slipped with a mounting medium to avoid fluorescence fading (Vectashield, Vector Laboratories^®^).

### Immunoreactivity quantification

Counts of the number of Fos-immunoreactive neurons were carried out at 20X magnification with an Olympus microscope (Model BX51) equipped with a digital camera. Images were generated for prelimbic cortex (PL), IL cortex, basal nucleus of the amygdala (BA), lateral portion of the central nucleus of the amygdala (CeL), which also included the intercalated cells (ITC), and the medial portion of the central nucleus of the amygdala (CeM). To be considered positive for Fos-like immunoreactivity, the nucleus of the neurons had to be of appropriate size (area ranging from 100 to 500 µm^2^) and shape (at least 50% of circularity), show brown-black staining of oxidized DAB-Ni, and be distinct from the background. Fos positive cells were automatically counted and averaged for each hemisphere at 2–3 distinct rostrocaudal levels of each structure (Metamorph software version 6.1). For prefrontal cortex sections, the antero-posterior levels were +2.7 mm, +3.2 mm and +3.7 mm from bregma. For amygdala sections, the antero-posterior levels were +2.3 mm and +2.8 mm from bregma. The density of Fos positive neurons was calculated by dividing the number of Fos positive neurons by the total area of each region.

BDNF images were obtained using the same microscope equipped with a fluorescent lamp (X-Cite^®^, Series 120 Q) and a digital camera, for the same structures quantified for Fos immunoreactivity. Image pairs were acquired at 20X magnification using the appropriate filter sets for green Alexa Fluor 488 or red Alexa Fluor 594 fluorescence, respectively for NeuN or BDNF labeling. Background luminescence for all images was digitally removed. The threshold was automatically adjusted and the percentage of overlapping area between NeuN and BDNF images (co-labeling) was determined.

### Statistical analysis

Statistical significance was determined with Student’s t-test (unpaired, two-tailed). The average of BDNF-NeuN overlapping area or Fos positive cells for each brain hemisphere was calculated and used for group comparisons. The level of statistical significance adopted was *p* < 0.05. All statistical analyses were performed using the Statistica software package (Version 6.0, Statsoft^®^, Tulsa, USA).

## Results

**Figure 1:**
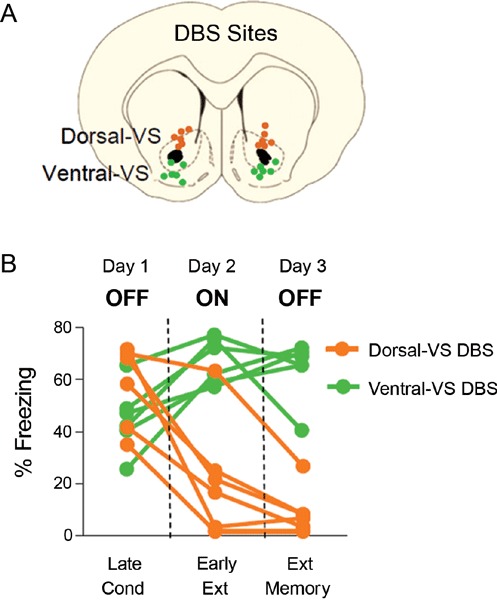
**DBS of the ventral striatum can either enhance or impair fear extinction, depending on the site of stimulation (modified from Rodriguez-Romaguera et al., 2012)**. **(A)** Placement of DBS electrode tips within the dorsal-VS (orange circles) and ventral-VS (green circles). **(B)** Individual data showing that DBS of dorsal-VS (*n* = 6) decreased fear expression on Day 2 (DBS ON) compared to Day 1 (DBS OFF), and enhanced extinction memory, as shown by the maintenance of low levels of freezing on Day 3 with DBS OFF. In contrast, DBS of ventral-VS (*n* = 6) increased fear expression on Day 2 (DBS ON) as compared to Day 1 (DBS OFF) and impaired extinction memory, as shown by the maintenance of high levels of freezing on Day 3 with DBS OFF. Data shown in blocks of two trials.

We previously reported that 3 hrs of continuous DBS (130 Hz) of dorsal-VS enhanced extinction memory, whereas the same duration of stimulation of ventral-VS impaired extinction memory (Rodriguez-Romaguera et al., 2012). Figure 1 shows electrode placements and behavioral data of individual rats from our previous study. As reported, rats receiving DBS of dorsal-VS on Day two showed reduced freezing at the start of extinction (DBS on), and reduced freezing during the extinction memory test on Day three (DBS off). In contrast, DBS of ventral-VS increased freezing on Day two and impaired extinction memory on Day three.

## DBS of the dorsal-VS, but not ventral-VS, increases Fos expression in PL and IL

**Figure 2:**
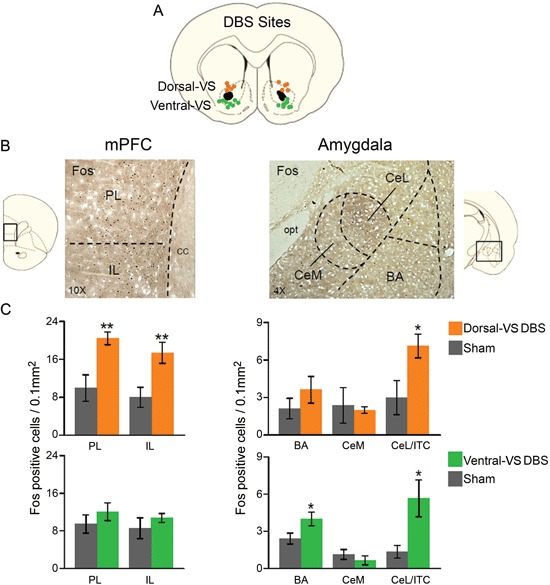
**DBS of dorsal-VS increases neuronal activity within extinction circuits. (A)** Placement of DBS electrode tips within the dorsal-VS (orange circles) and ventral-VS (green circles). **(B)** Representative micrographs showing Fos labeled neurons in prelimbic (PL) and infralimbic (IL) regions of medial prefrontal cortex (mPFC, 10x magnification, left), and the lateral portion of the central nucleus of the amygdala, including the intercalated cells (CeL/ITC), in rats administered DBS in the dorsal-VS (Amygdala, 4x magnification, right). **(C)** DBS of dorsal-VS increased Fos expression in PL, IL and CeL/ITC, but not in BA or CeM (Sham, *n* = 5; DBS of dorsal-VS, *n* = 4). In contrast, DBS of ventral-VS increased Fos in the basal nucleus of the amygdala (BA) and CeL/ITC, but not in PL, IL or CeM (Sham, *n* = 8; DBS of ventral-VS, *n* = 8). Legend: CeM = medial portion of the central nucleus of the amygdala, cc = corpus callosum, opt = optic tract. Data shown as mean and SEM. *p < 0.05 **p < 0.01.

In a different set of rats, we compared the effects of 3 hrs of continuous DBS in the dorsal- or ventral-VS on the expression of Fos protein in the PL and IL subregions of the mPFC, as well as in different amygdala subnuclei: the basal nucleus (BA), CeL, which also included ITC, CeM. As illustrated in Figure 2, DBS of the dorsal-VS significantly increased the number of Fos positive neurons in PL [Sham: 10; DBS of dorsal-VS: 20; *t*_(16)_ = −3.13; *p* = 0.007], IL [Sham: 8; DBS of dorsal-VS: 17; *t*_(16)_ = −3.02; *p* = 0.008], and CeL/ITC [Sham: 3; DBS of dorsal-VS: 7; *t*_(14)_ = −2.48; *p* = 0.03]. No significant differences were observed in the number of Fos positive neurons in BA [Sham: 2; DBS of dorsal-VS: 4; *t*_(14)_ = −1.12; *p* = 0.28] or CeM [Sham: 2; DBS of dorsal-VS: 2; *t*_(14)_ = 0.26; *p* = 0.80].

In contrast to dorsal-VS, DBS of ventral-VS did not alter Fos expression in PL [Sham: 9; DBS of ventral-VS: 12; *t*_(30)_ = −0.98; *p* = 0.34], IL [Sham: 9; DBS of ventral-VS: 11; *t*_(30)_ = −0.90; *p* = 0.38], or CeM [Sham: 1; DBS of ventral-VS: 1; *t*_(27)_ = 0.89; *p* = 0.38]. However, a significant increase in Fos positive neurons was observed in both BA [Sham: 2; DBS of ventral-VS: 4; *t*_(27)_ = −2.20; *p* = 0.04] and CeL/ITC [Sham: 1; DBS of ventral-VS: 6; *t*_(27)_ = −2.67; *p* = 0.01, see Figure 2]. Thus, using Fos expression as an indicator of neuronal activity, DBS of dorsal-VS increased activity in PL and IL, whereas DBS of ventral-VS increased activity in BA. DBS of either VS site increased activity in CeL/ITC.

## DBS of the dorsal-VS, but not ventral-VS, increases BDNF expression in PL and IL

**Figure 3:**
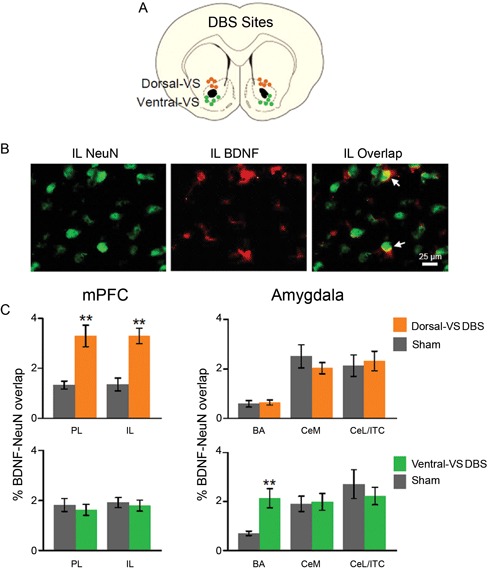
**DBS of dorsal-VS increases neuronal BDNF in PL and IL. (A)** Placement of DBS electrode tips within the dorsal-VS (orange circles) and ventral-VS (green circles). **(B)** Representative micrographs showing IL labeling of neuronal marker NeuN (left), BDNF (middle), and BDNF-NeuN overlap (right, white arrows). **(C)** DBS of dorsal-VS increased BDNF-NeuN overlap in PL and IL subregions of the mPFC, but not in the amygdala (Sham, *n* = 4; DBS of dorsal-VS, *n* = 6). In contrast, DBS of ventral-VS increased BDNF-NeuN overlap in BA, but not in PL, IL, CeM or CeL/ITC (Sham, *n* = 5; DBS of ventral-VS, n = 5). Legend: CeM = medial portion of the central nucleus of the amygdala, CeL = lateral portion of the central nucleus of the amygdala, ITC = intercalated cells. Data shown as mean and SEM. **p < 0.01.

Similar to Fos expression, levels of neuronal BDNF in mPFC and amygdala were altered by DBS. As illustrated in Figure 3, DBS of the dorsal-VS significantly increased the percentage of overlap (co-labeling) between BDNF and the neuronal marker NeuN in both PL [Sham: 1.3%; DBS of dorsal-V: 3.3%; *t*_(18)_ = −3.57; *p* = 0.002] and IL [Sham: 1.4%; DBS of dorsal-VS: 3.3%; *t*_(18)_ = −4.52; *p* < 0.001]. Notably, no group differences in BDNF-NeuN overlap were observed in BA [Sham: 0.6%; DBS of dorsal-VS: 0.6%; *t*_(18)_ = −0.30; *p* = 0.77], CeM [Sham: 2.5%; DBS of dorsal-VS: 2.0%; *t*_(18)_ = 1.02; *p* = 0.32], or CeL/ITC [Sham: 2.1%; DBS of dorsal-VS: 2.3%; *t*_(18)_ = −0.30; *p* = 0.77].

In contrast to dorsal-VS, DBS of the ventral-VS did not alter the neuronal levels of BDNF in PL [Sham: 1.8%; DBS of ventral-VS: 1.6%; *t*_(18)_ = 0.57; *p* = 0.58] and IL [Sham: 1.9%; DBS of ventral-VS: 1.8%; *t*_(18)_ = 0.40; *p* = 0.70]. In addition, no changes in BDNF levels were observed in CeM [Sham: 1.9%; DBS of ventral-VS: 2.0%; *t*_(18)_ = −0.19; *p* = 0.85] and CeL/ITC [Sham: 2.7%; DBS of ventral-VS: 2.2%; *t*_(18)_ = 0.71; *p* = 0.48]. However, DBS of ventral-VS significantly increased the neuronal levels of BDNF in BA [Sham: 0.7%; DBS of ventral-VS: 2.1%; *t*_(18)_ = −3.56; *p* = 0.002, see Figure 3], in agreement with Fos expression. In fact, all the areas that showed increased Fos also showed increased BDNF, with the exception of CeL/ITC which showed increased Fos but no increase in BDNF.

## Discussion

**Figure 4:**
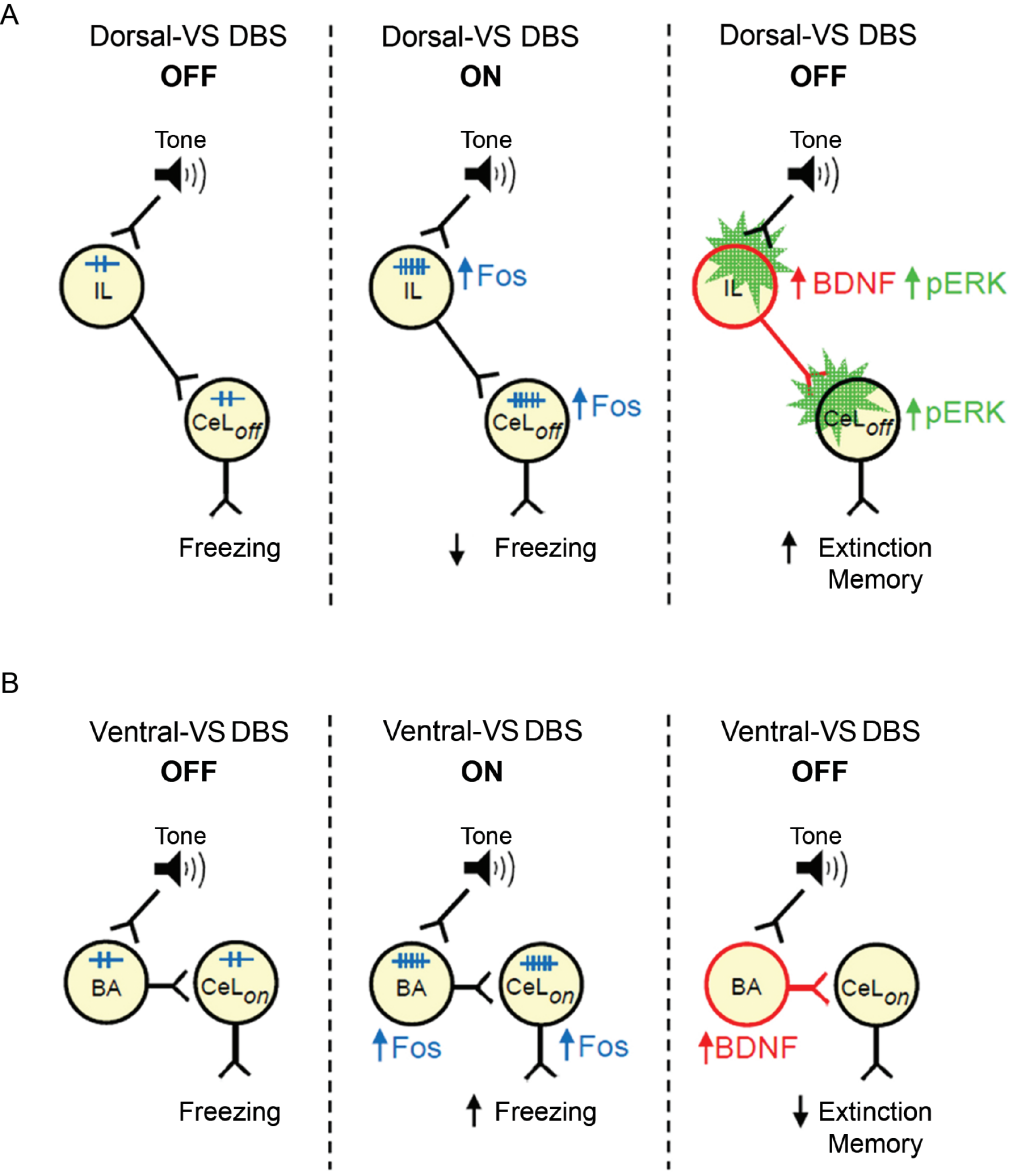
**Suggested models of how DBS of dorsal-VS and ventral-VS affect fear. (A)** Middle: DBS of dorsal-VS (ON) increases neuronal activity (Fos) in the IL-CeL_off_ circuit, decreasing freezing to a conditioned tone. Right: DBS of dorsal-VS also increases BDNF in IL and induces plasticity (pERK) in IL and CeL_off_ (Rodriguez-Romaguera et al., 2012), thereby enhancing extinction memory in the absence of DBS. **(B)** Middle: DBS of ventral-VS increases neuronal activity (Fos) in the BA-CeL_on_ circuit, increasing freezing to a conditioned tone. Right: DBS of ventral-VS also increases BDNF in BA, thereby impairing extinction memory in the absence of DBS.

Following up on our study of DBS in dorsal-VS and ventral-VS (Rodriguez-Romaguera et al., 2012), we used an immunocytochemical approach to uncover possible mechanisms of DBS effects on fear expression and extinction memory. We found that DBS of dorsal-VS increased expression of Fos and neuronal BDNF in both PL and IL subregions of the mPFC. In contrast, DBS of ventral-VS increased the expression of Fos and BDNF only in BA. Increased Fos expression in CeL/ITC was observed after DBS of both dorsal-VS or ventral-VS. Our data suggest that enhanced extinction memory observed with DBS of dorsal-VS may be due to increased BDNF levels in IL neurons, leading to increased activation of inhibitory neurons in CeL/ITC (Figure 4).

Recent findings suggest that PL and IL cortices have opposite effects on fear responses (Vidal-Gonzalez et al., 2006; Laurent and Westbrook, 2009; Sierra-Mercado et al., 2011). PL sends excitatory projections to BA (Vertes, 2004; Likhtik et al., 2005), a region necessary for fear expression (Anglada-Figueroa and Quirk, 2005), whereas IL projects to GABAergic (gamma-aminobutyric acid) cells in CeL, which inhibits CeM outputs and consequently fear expression (Royer and Pare, 2002; Quirk et al., 2003; Amano et al., 2010). It was somewhat surprising, therefore, that dorsal-VS DBS increased Fos expression in PL as well as IL. Optogenetic activation of IL pyramidal neurons has been shown to reduce PL activity through feed-forward inhibition (Ji and Neugebauer, 2012). Therefore, increased Fos in PL may reflect augmented GABAergic activity in PL. In addition, extinction training increases Fos expression in both PL and IL, as well as CeL/ITC (Santini et al., 2004; Knapska and Maren, 2009; Kim et al., 2010; Plendl and Wotjak, 2010), similar to what we observed for DBS of dorsal-VS. In contrast, DBS of ventral-VS increased Fos expression in BA, consistent with increased fear expression (Amano et al., 2011; Sangha et al., 2013). BA projections to VS terminate in the ventral-VS, rather than the dorsal-VS (Kelley et al., 1982; Mcdonald, 1991), suggesting that increased BA activity may be due to antidromic activation of BA fear neurons (Herry et al., 2008), by DBS of ventral-VS. Surprisingly, DBS of ventral-VS also increased Fos expression in CeL/ITC, suggesting that DBS at this site may be activating a different population of CeL neurons mediating fear expression (Ciocchi et al., 2010). Alternatively, both PL and IL project through the VS to reach the amygdala (St Onge et al., 2012). Thus, stimulation of distinct prefrontal-amygdalar fibers passing through the VS could explain the opposite effects of adjacent DBS sites, however more studies are needed to address this possibility.

Previous studies have shown that extinction training increases BDNF gene expression in the mPFC (Bredy et al., 2007), and infusion of BDNF into IL facilitates extinction learning (Peters et al., 2010). Furthermore, a common polymorphism in the BDNF gene has been associated with deficits in extinction memory in both mice and humans (Soliman et al., 2010). In particular, the same polymorphism was associated with reduced NMDA-glutamatergic transmission specifically in IL (Pattwell et al., 2012). Therefore, the increase in IL BDNF following DBS of dorsal-VS, but not ventral-VS, may mediate the enhancement of fear extinction by DBS. DBS of dorsal-VS also increased BDNF levels in PL, however prior studies have demonstrated that extinction is unaffected by intra-PL infusion of BDNF (Rosas-Vidal et al., 2012), or deletion of the BDNF gene in PL (Choi et al., 2010).

In contrast to DBS of dorsal-VS, DBS of ventral-VS modified BDNF in BA, but not in prefrontal cortex. Previous studies have shown that BDNF levels in BA are significantly increased after fear conditioning (Rattiner et al., 2004; Ou and Gean, 2006). In addition, blockade of BDNF signaling in the BA disrupted acquisition of conditioned fear (Rattiner et al., 2004), and expression of BDNF in this area is necessary for maintenance of fear memories (Ou et al., 2010). Therefore, increased BDNF levels in BA could contribute to augmented fear memory with DBS of ventral-VS, leading to impaired fear extinction memory in the next day.

Extinction-based therapy is currently among the most effective treatments for OCD (Rasmussen and Eisen, 1997; Franklin and Foa, 2011; Olatunji et al., 2013), and DBS of the VC/VS increases the effectiveness of such therapies (Denys et al., 2010; de Koning et al., 2011). Serum levels of BDNF are reduced in OCD (Maina et al., 2010; Fontenelle et al., 2012), and a BDNF polymorphism is correlated with impaired response to extinction-based therapy (Fullana et al., 2012), suggesting a role of BDNF in OCD pathophysiology. OCD patients show impaired fear extinction and reduced activity in the vmPFC, a homologue of rodent IL (Milad et al., 2013). Thus, DBS-induced increases in prefrontal BDNF, as well as prefrontal monoamines (van Dijk et al., 2012), suggests that DBS of the VC/VS in OCD patients may repair faulty prefrontal circuits (Figee et al., 2013), thereby improving extinction-based therapy.

## Conflict of interest statement

The authors declare that the research was conducted in the absence of any commercial or financial relationships that could be construed as a potential conflict of interest.
